# A multiple alignment workflow shows the effect of repeat masking and parameter tuning on alignment in plants

**DOI:** 10.1002/tpg2.20204

**Published:** 2022-04-13

**Authors:** Yaoyao Wu, Lynn Johnson, Baoxing Song, Cinta Romay, Michelle Stitzer, Adam Siepel, Edward Buckler, Armin Scheben

**Affiliations:** ^1^ Institute for Genomic Diversity Cornell Univ. Ithaca NY 14853 USA; ^2^ Agricultural Genomics Institute at Shenzhen Chinese Academy of Agricultural Sciences Shenzhen China; ^3^ USDA‐ARS Ithaca NY 14853 USA; ^4^ Simons Center for Quantitative Biology Cold Spring Harbor Laboratory Cold Spring Harbor NY 11724 USA; ^5^ Dep. of Molecular Biology and Genetics Cornell Univ. Ithaca NY 14853 USA

## Abstract

Alignments of multiple genomes are a cornerstone of comparative genomics, but generating these alignments remains technically challenging and often impractical. We developed the *msa_pipeline* workflow (https://bitbucket.org/bucklerlab/msa_pipeline) to allow practical and sensitive multiple alignment of diverged plant genomes and calculation of conservation scores with minimal user inputs. As high repeat content and genomic divergence are substantial challenges in plant genome alignment, we also explored the effect of different masking approaches and parameters of the LAST aligner using genome assemblies of 33 grass species. Compared with conventional masking with RepeatMasker, a masking approach based on *k*‐mers (nucleotide sequences of *k* length) increased the alignment rate of coding sequence and noncoding functional regions by 25 and 14%, respectively. We further found that default alignment parameters generally perform well, but parameter tuning can increase the alignment rate for noncoding functional regions by over 52% compared with default LAST settings. Finally, by increasing alignment sensitivity from the default baseline, parameter tuning can increase the number of noncoding sites that can be scored for conservation by over 76%. Overall, tuning of masking and alignment parameters can generate optimized multiple alignments to drive biological discovery in plants.

## Core Ideas

- We developed a practical multiple alignment pipeline for sensitive alignment of plant genomes.
- Repeat masking based on *k*‐mers rather than repeat libraries increased alignment of functional regions.
- Parameter tuning substantially boosted alignment rates of noncoding functional regions.

**Abbreviations**
BOP: grass lineage including subfamilies Bambusoideae, Oryzoideae, and Pooideae
CDS: coding sequence
GC: guanine‐cytosine
GERP: Genomic Evolutionary Rate Profiling
mya: million years ago
PACMAD: grass lineage including subfamilies Panicoideae, Aristidoideae, Chloridoideae, Micrairoideae, Arundinoideae, and Danthonioideae
RED: REpeat Detector
RS: rejected substitution.

## 1 INTRODUCTION

Multiple sequence alignment is a key challenge in comparative genomics and evolutionary studies (Armstrong et al., 2019; Chowdhury & Garai, 2017). As the number of novel genomes being generated is rapidly accelerating, researchers rely on robust tools that can scale from dozens to hundreds of genomes. Many tools are available for pairwise or multiple alignment of genome sequences (Armstrong et al., 2020; Frith & Kawaguchi, 2015; Marçais et al., 2018; Minkin & Medvedev, 2020). However, these tools generally require a range of inputs such as a phylogenetic tree and repeat masking information. Pairwise alignment tools such as LASTZ and LAST also need their outputs to be postprocessed before subjecting them to multiple alignment using a different tool. In addition, many tools do not scale well to large sets of plant genomes. The many requirements and types of software involved can make the seemingly straightforward task of multiple sequence alignment technically challenging for individual researchers. In this work, we therefore developed the practical *msa_pipeline* to generate multiple sequence alignments from a reference genome and a set of query genomes. The *msa_pipeline* relies on the LAST aligner and aims to minimize the amount of user effort required to rapidly produce a high‐quality multiple alignment of diverged plant genomes. We tested the computational efficiency of the pipeline and the effect of a range of repeat masking and alignment parameters using public grass genome sequences and compared the pipeline with the Cactus aligner. Overall, we present the publicly available *msa_pipeline* and recommend repeat masking and alignment strategies that enhance alignment of genic and intergenic regions of diverged plant genomes.

## 2 METHODS

### 2.1 Selection of syntenic regions for alignment analysis

Whole genome alignment is computationally demanding (Supplemental Table S1). To accelerate comparison of multiple alignments constructed with a range of parameters, we used a subset of genomic sequences from our target species. Specifically, we used MCScan (Wang et al., 2012) to select two syntenic regions that are common to grass genomes and contain 100 genes each based on the Ensembl Sorghum_bicolor_NCBIv3 annotation (see Supplemental Data S1 and S2). We refer to these syntenic sequences as “mini‐genomes.” We aimed to compare alignment results for these mini‐genomes from two distinct clades of grasses known as BOP (subfamilies Bambusoideae, Oryzoideae, and Pooideae) and PACMAD (subfamilies Panicoideae, Aristidoideae, Chloridoideae, Micrairoideae, Arundinoideae, and Danthonioideae). The reference genome used for the 18 selected BOP species was rice (*Oryza sativa* L.) version IRGSP‐1.0, and the reference genome for the 14 selected PACMAD species was maize (*Zea mays* L.) version B73V4. Sequences were obtained from GenBank. The guanine and cytosine content ranged from 33 to 46% in BOP and 37 to 46% in PACMAD (Supplemental Data S3). *Oryza longistaminata* was excluded from further analyses due to poor alignment rates.

### 2.2 Repeat masking approaches

Repeats often cannot be aligned accurately between genomes. For this reason, repetitive sequences are often replaced with “N”s (hard‐masked) or set to lowercase (soft‐masked) and treated differently from nonrepetitive sequences during alignment. Aligners will generally ignore hard‐masked sequence but can use soft‐masked sequence, for instance to extend alignments that were initiated in unmasked regions. To identify repeats, repeat detection methods such as the widely used RepeatMasker generally rely on libraries of repeat elements that are aligned to genomes to identify known repeats. Here, we used public RepeatMasker annotations available via GenBank. A drawback of RepeatMasker annotations is that repeat elements not similar to those in the repeat library will not be masked and, conversely, nonrepetitive functional elements with similarities to repeats may be erroneously masked (Bayer et al., 2018). To compare *k*‐mer based masking approaches to RepeatMasker, we therefore also conducted masking with the *k*‐mer based approach REpeat Detector (RED) (Girgis, 2015) and a novel *k*‐mer based approach (Song et al., 2020) that we hereafter refer to as “KMER.” The KMER approach is similar to the approach used in RED, relying on generation of the *k*‐mer frequency spectrum and a second derivative test to determine a frequency cut‐off for a class of high‐frequency *k*‐mers that are likely to be repeats (https://github.com/baoxingsong/dCNS). However, KMER, unlike RED, does not use further classification based on a trained model for repeat identification. KMER is thus a more conservative repeat detection approach, which, unlike RepeatMasker and RED, will not mask low‐copy repeats. KMER is therefore less sensitive than other methods, but a potential advantage of KMER for preprocessing genomes for alignment is that it has less potential for false positives caused by bias through training data or lineage‐specific repeat databases.

**Core Ideas**

- We developed a practical multiple alignment pipeline for sensitive alignment of plant genomes.
- Repeat masking based on *k*‐mers rather than repeat libraries increased alignment of functional regions.
- Parameter tuning substantially boosted alignment rates of noncoding functional regions.

### 2.3 Sampling the alignment parameter space

We performed multiple alignment with the *msa_pipeline* for PACMAD clade species and BOP clade species using three sets of differently masked sequences (RepeatMasker, RED, KMER) for each clade. Each masking approach was furthermore tested with hard‐masked and soft‐masked sequences. We varied 10 LAST pairwise alignment parameters to explore the parameter space, including parameters controlling gap/mismatch penalty sizes, number of initial matches, and simple repeat masking. A total of 750 parameter combinations were randomly sampled from the parameter space.

Two custom substitution penalty matrices (RETRO and RETRO SIMPLE; see Supplemental Data S4) were generated based on observed substitution rates in aligned maize retrotransposons. Briefly, we used MAFFT alignments of 5′ and 3′ long terminal repeats of individual retrotransposon copies from Stitzer et al. (2019) to count base substitutions that have accumulated since the transposable element inserted, using the seg.sites function implemented in ape v5.4 (Paradis & Schliep, 2018). This provides an empirical measure of substitution rates in maize, reflecting the high rate of transitions.

### 2.4 Evaluation of alignments

We focused on the alignment of functional elements of the genome as a measure of alignment quality. We used a broad definition of these functional elements, including noncoding functional regions (gene promoters, untranslated regions, introns, open chromatin) and coding sequence (CDS). Promoters were defined as the 1 kb region upstream of each gene. Functional elements were identified based on reference genome annotations for maize (for the PACMAD clade) and rice (for the BOP clade) as well as publicly available open chromatin data (Joly‐Lopez et al., 2020; Ricci et al., 2019; Zhou et al., 2021). We defined *a* as the number of bases of reference functional elements with at least half of the query species aligned, while *e* is defined as the total number of bases of reference functional elements.

(1) $$\mathrm{Recall}\;=\frac{a}{e}$$

Thus, we define approximate alignment recall as shown in Equation 1.

(2) $$\mathrm{Precision}\;=\frac{a}{a+n}$$

In Equation 2, we defined the number of aligned nonfunctional intergenic bases as n and use them to help calculate approximate alignment precision. A key assumption here is that intergenic regions distant from genes and with inaccessible chromatin are enriched for erroneous alignments compared with our defined functional regions. This assumption is a caveat for our calculation of precision because false positives are identified based on this assumption rather than a ground truth.

(3) $$F_{1}\;=\;\frac{2}{\left(\frac{1}{\mathrm{Recall}}+\frac{1}{\mathrm{Precision}}\right)}$$

Finally, we calculated the F_1_ score (harmonic mean of precision and recall) using our calculations of alignment recall and precision.

To compare the results from our pipeline to an alternative state‐of‐the‐art alignment pipeline designed for interspecies alignment, we conducted alignments with the Cactus 1.2.3 aligner (Armstrong et al., 2020), which we hereafter refer to as “Cactus.” Cactus is a powerful genome alignment pipeline that relies on the LASTZ aligner and additional alignment refinement steps. Cactus has been used particularly to produce highly complete alignments of animal genomes (e.g., Feng et al., 2020) but is highly computationally demanding and allows limited tuning of alignment parameters, which may hamper its use for alignment of large and highly divergent plant genomes.

### 2.5 Alignments affect the detection of genomic conservation

To assess how the alignment affects the inference of genomic conservation, we calculated conservation using Genomic Evolutionary Rate Profiling (GERP) (Davydov et al., 2010) with the *msa_pipeline* in the PACMAD and BOP clade, respectively. For each alignment generated from the 750 parameter combinations, we used a fixed neutral tree and considered all sites with rejected substitution (RS) scores greater than 80% of the maximum RS score to be conserved. The threshold for considering a site conserved in BOP was RS = 1.568 and the threshold in PACMAD was RS = 1.072.

To further explore the site‐to‐site alignment, we compared the Pearson's correlation of GERP RS scores between the PACMAD and BOP clades. We expect a substantial proportion of conservation to be clade‐specific and thus uncorrelated, limiting the maximum correlation possible. However, we cautiously consider an increase in correlation as a potential indicator for improvements in alignment of functional sequences conserved across grass clades. We used LAST alignment to lift‐over genomic coordinates between the rice genome (the reference for BOP) and the maize genome (the reference for PACMAD). For the sites that could be lifted over between rice and maize, we then calculated the correlation of GERP RS scores between PACMAD and BOP across the genome and for different functional genomic regions.

## 3 RESULTS AND DISCUSSION

### 3.1 Features and implementation of *msa_pipeline*

**FIGURE 1:**
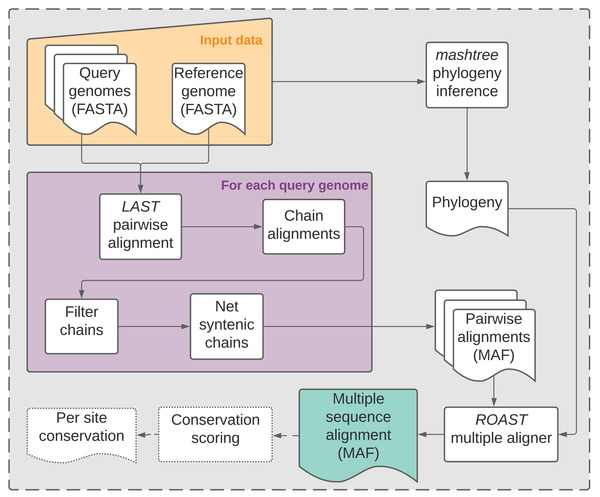
Schema describing the snakemake *msa_pipeline* for multiple sequence alignment. The pipeline uses a set of genome sequences in FASTA format as input, generating pairwise alignments to a reference genome with the LAST aligner (or another supported aligner) and then processing and combining these alignments into a multiple alignment using roast. Optionally, users can then call the observed per‐site conservation based on comparison to the expected conservation under a neutral model calculated by the pipeline. MAF, multiple alignment format

The *msa_pipeline* only requires a set of masked genomes in FASTA format as input, outputting a multiple sequence alignment in multiple alignment format (Figure 1). Dependencies are handled using conda environments and snakemake is deployed as a workflow manager. For pairwise alignment, we focus on the LAST alignment tool, because the sensitivity of LAST makes it highly suitable for comparison of diverged interspecies genomes (Frith & Noé, 2014). High sensitivity is important for many downstream analyses of the alignment because it facilitates alignment of functional sequences such as promoters and enhancers that are located in more variable intergenic regions. For convenience, we also support use of minimap2 (Li, 2018) and GSAlign (Lin & Hsu, 2020) for alignments of less diverged genomes. Pairwise alignment to the reference genome can be conducted in parallel, with the main pipeline bottleneck being multiple sequence alignment using the single‐threaded ROAST program (https://github.com/multiz/multiz). The pipeline outputs multiple alignments in multiple alignment format . We further provide an optional step in the pipeline to use the multiple alignment to generate per‐site genome‐wide conservation scores calculated with GERP (Davydov et al., 2010) and phyloP (Pollard et al., 2010) based on a neutral model generated with phyloFit from the phast package (Siepel et al., 2005). The runtime and memory usage of *msa_pipeline* is low at default settings (Supplemental Table S1).

### 3.2 Selecting alignment metrics for benchmarking and improving multiple alignment in plant genomes

Measuring the accuracy of alignments between distantly related species is challenging because ground‐truth alignments are generally unknown. Studies have therefore measured alignment accuracy by focusing on partial alignments of conserved functional sequences such as exons (Frith et al., 2010; Sharma & Hiller, 2017) or by relying on simulated sequences (Armstrong et al., 2020). To reduce biases caused by simulation parameters or by an exclusive focus on CDS, we measured accuracy based on alignments of functional sequences in coding and noncoding regions. Specifically, we calculated precision, recall, and F_1_ score of functional regions, assuming that alignments of nonfunctional regions were false positives (see Methods). Although this simplifying assumption is unlikely to generally be the case, the resulting approximate measures are useful for benchmarking alignment quality in the functional regions of the genome that are most important for the majority of downstream analyses.

### 3.3 Appropriate repeat masking can improve multiple alignment performance

A major obstacle to accurate and efficient alignment is the large proportion of repetitive sequence found in most plant genomes. In contrast to masking tools like RepeatMasker that rely on repeat databases, approaches such as RED (Girgis, 2015) or KMER (Song et al., 2020) try to avoid database bias by using repetitive *k*‐mers in the genome to identify repeats. Here, we compared RepeatMasker, RED, and KMER and tested their effect on subsequent multiple sequence alignment in grasses. We selected species from the PACMAD grass clade, which diverged ∼30 million years ago (mya) (Cotton et al., 2015), as well as species from the BOP grass clade, which diverged ∼50 mya (Christin et al., 2014). Although conservation of regulatory sequence between BOP and PACMAD may be relatively low (Guo et al., 2003), a recent study of plant regulatory element conservation suggests that over half of accessible chromatin regions remain conserved at divergence times of 24 mya (Lu et al., 2019). We thus expect considerable conservation of non‐CDS within the species selected from the BOP and PACMAD clades.

**FIGURE 2:**
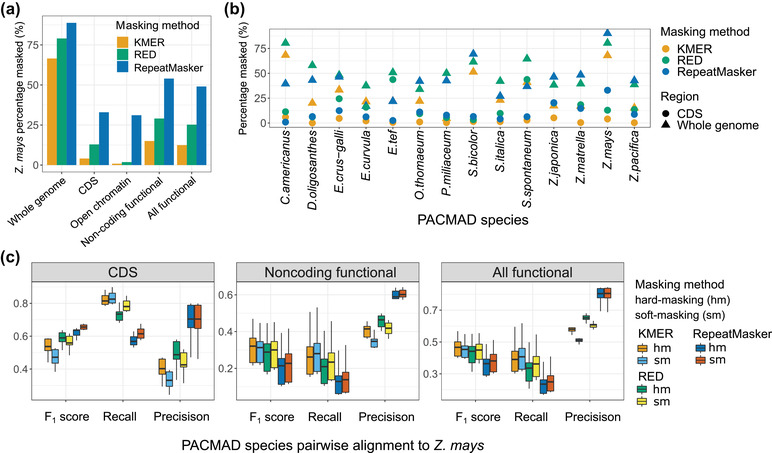
Effect of repeat masking methods on alignment of functional genomic regions. (a) Comparison of the masking rate for different genomic regions in maize using three masking methods. The RepeatMasker method masks substantially larger proportions of coding and functional sequence than the *k*‐mer based methods (REpeat Detector [RED] and KMER). (b) The masking rate for the whole genome and for CDS shown in 14 species of the PACMAD grass clade. (c) Boxplots of pairwise alignment performance (see Methods) of 13 species of the PACMAD clade aligned to maize. Hard‐masked alignments perform similarly to soft‐masked alignments. The F_1_ scores (harmonic mean of precision and recall) indicate that for noncoding sequence *k*‐mer based methods provide a better trade‐off between alignment precision and recall. CDS, coding sequence

We found substantial differences between all three masking methods, affecting the amount of putative false positive masking in coding, open chromatin regions and noncoding functional regions (see Methods for definition of these regions). In maize, compared to KMER, RepeatMasker masked an additional 28.89% of CDS and 38.96% of noncoding functional regions (Figure 2a, Supplemental Table S2). The overall genome‐wide repeat content of 88% estimated by RepeatMasker is broadly in line with the ∼80% repeat content reported in the literature (Springer et al., 2018; Sun et al., 2018). In contrast, the 66% repeat content estimated by KMER is substantially lower than reports in the literature, indicating underestimation of the full repeat content. KMER also failed to mask substantial numbers of repeats in fragmented genome assemblies such as those of *Dichanthelium oligosanthes*, *Panicum miliaceum*, and *Eragrostis tef* (Supplemental Data S3). Furthermore, KMER masked considerably less sequence than RED (Figure 2a, Supplemental Tables S2 and S3). Despite providing inaccurate estimates of total repeat content, KMER displayed the most favorable trade‐off between the masking rate and the rate of masked coding and noncoding functional sequence across most genomes (Figure 2b, Supplemental Figure S1, and Supplemental Results).

Based on analysis in the PACMAD clade, genomes masked with KMER produced sensitive alignments (mean F_1 _= 0.4670 for pairwise alignment; multiple alignment F_1 _= 0.4809) with higher alignment rates of functional sequence than those masked with RepeatMasker (mean F_1 _= 0.3569 for pairwise alignment; multiple alignment F_1 _= 0.3686) and those masked with RED (mean F_1 _= 0.4284 for pairwise alignment; multiple alignment F_1 _= 0.4506) (Figure 2c; see Supplemental Data S5 and S6). KMER and RED each show significantly higher F_1_ scores than RepeatMasker (Student's *t*‐test, one‐sided, *p *< .05; Supplemental Table S4), however KMER does not have a significantly higher F_1_ score than RED (Student's *t*‐test, one‐sided, *p *= .07). Similar analyses in the BOP clade were consistent with these results (Figures S1C and S1D). These results are also in line with those reported in a recent study that suggested RED *k*‐mer masking is preferable compared with library‐based approaches for efficient masking of plant genomes prior to whole genome alignment (Contreras‐Moreira et al., 2021). Overall, our findings suggest that using *k*‐mer based masking improves alignment, with hard‐masking performing comparable with soft‐masking (Supplemental Table S4) while also providing minor improvements in runtime (Supplemental Tables S1 and S5).

### 3.4 Exploration of alignment parameter space shows potential for improving intergenic alignment rates

Alignment parameters such as substitution matrices and gap penalties can have a substantial effect on alignment (Frith, Hamada, & Horton, 2010). Often default alignment settings are based on testing in mammalian genomes that are less repetitive and diverse than those of many plants. To explore the alignment parameter space for grass genomes, we tested 750 different combinations of 10 LAST parameters including gap penalties and substitution matrices for multiple alignments (Supplemental Table S6). By approximating recall and precision as measures of alignment performance, we assessed 750 differently parametrized multiple alignments of syntenic regions spanning 100 genes each (referred to here as mini‐genomes) in the grass clades known as PACMAD and BOP (Figures S2–S6). We found that some of the best alignments were generated using default LAST alignment parameters (recall = 0.2823, precision = 0.9095, F_1_ = 0.4309) and Cactus alignment (recall = 0.4040, precision = 0.8478, F_1_ = 0.5472). As expected based on higher coding region conservation, coding regions (recall = 0.48‐0.78; precision = 0.47‐0.99) showed substantially higher recall than noncoding regions (recall = 0.02‐0.39; precision = 0.63‐0.93, Supplemental Table S7, and Supplemental Data S7).

**FIGURE 3:**
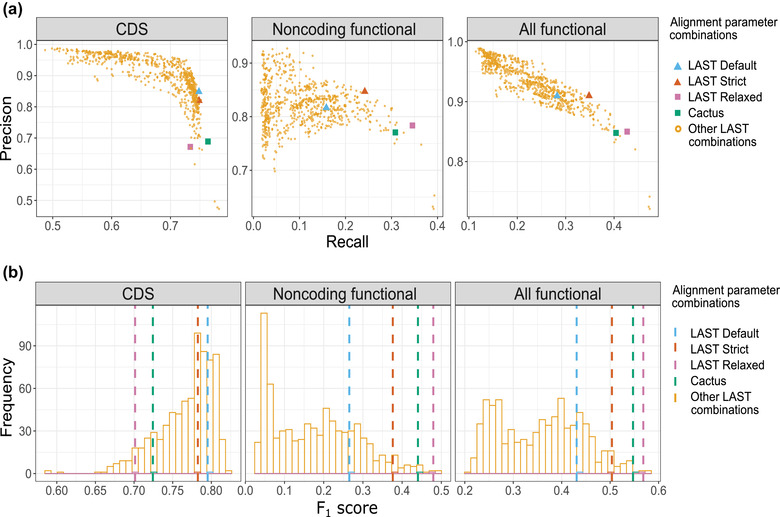
Multiple alignment performance of 750 LAST parameter combinations for regions syntenic to the sorghum mini‐genome sequence (see Methods) in the PACMAD grass clade. Tested parameter combinations are compared to the alignment performance of default LAST parameters and the Cactus aligner based on (a) recall and precision as well as the (b) F_1_ score (harmonic mean of precision and recall). The LAST relaxed parameter set performs similarly to Cactus, improving alignments particularly in noncoding regions, while Last Default parameters outperform in coding sequence alignments. CDS, coding sequence

More interestingly, nondefault LAST parameter combinations showed substantial differences including some improvements in noncoding region alignment performance compared with the default parameters and Cactus. The default LAST penalty matrix and parameters favor precision over recall, which we found leads to low alignment rates in intergenic regions for divergent genomes like those in the PACMAD grass clade. In this study, we selected the parameter combination LAST strict (Supplemental Table S8) that shows equal precision compared with LAST default parameters but a recall of 0.35, corresponding to a 23% increase from the default (Supplemental Table S9). This gain in recall is mainly attributable to use of the HOXD70 penalty matrix and lower penalization of alignment gaps (Supplemental Table S8). The parameter combination LAST relaxed (Supplemental Table S8) further decreases the gap existence cost (parameter*‐a*), elevating the recall to 0.57 while maintaining a precision over 0.85. This parameter combination produces an alignment with similar precision and recall compared with the Cactus alignment in both the PACMAD and BOP clade (Figure 3, Supplemental Figure S5, Supplemental Tables S9 and S10, and Supplemental Data S7 and S8).

The parameter exploration analysis for the second mini‐genome recapitulated these differences between Cactus and the default as well as the selected LAST parameters (Supplemental Figure S6 and Supplemental Data S9). To ensure that parameter choices were not biased by the mini‐genome regions selected, we also evaluated the performance of the default parameters and LAST strict and LAST relaxed using whole genome alignments for each species of the PACMAD clade to the maize genome (Supplemental Figure S7 and Supplemental Data S10). This analysis supported the findings based on the mini‐genomes, showing that F_1_ scores followed the pattern relaxed > strict > default with significant differences between each group (Student's *t*‐test, one‐sided, *p *< .05; Supplemental Table S10). The LAST relaxed parameters also consistently generated higher F_1_ scores than the Cactus aligner across all functional regions, though Cactus had a greater F_1_ score in coding regions (Supplemental Table S9). These results could also be confirmed when using sorghum as the reference genome and when aligning potato to tomato (Supplemental Table S10), suggesting that they are broadly valid.

### 3.5 Multiple alignment parametrization facilitates detection of genomic conservation

**FIGURE 4:**
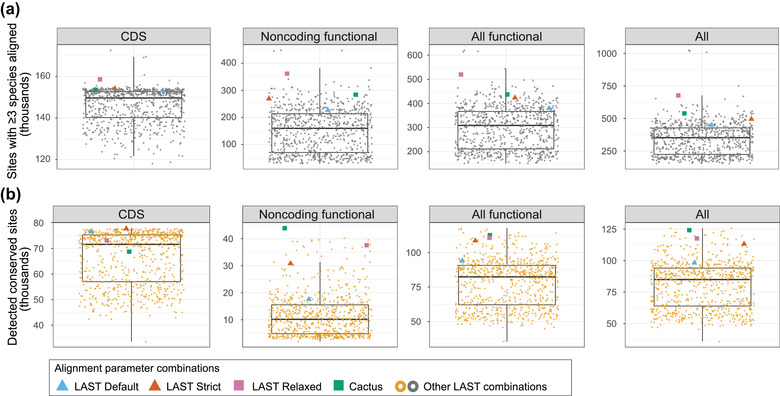
Alignment parameters affect conservation scoring in the PACMAD grass clade. A total of 750 LAST parameter combinations including the Default combination and the optimized strict and relaxed combinations (Supplemental Table S8) as well as a separate alignment using the Cactus aligner were compared by aligning a syntenic mini‐genome region spanning 100 genes of sorghum chromosome 3 (see Methods). (a) The number of sites with sufficient alignment depth (> = 3 species) to be scored for conservation in different genomic regions in the PACMAD grass clade. The LAST relaxed parameter combination substantially increases the number of sites that can be scored compared to Cactus and other LAST parameter combinations. (b) The number of conserved sites detected in different genomic regions in the PACMAD grass clade is affected by alignment parameters. The higher alignment rates provided by Cactus and LAST relaxed alignments are linked to a higher number of overall conserved sites detected, particularly in noncoding sequences. CDS, coding sequence

To evaluate how much the multiple alignment affects estimates of genomic conservation, we calculated the GERP conservation score based on the previously introduced 750 alignments of PACMAD and BOP generated with different alignment parameter combinations. In PACMAD, the number of sites that had sufficient alignment depth (≥3 species) to produce a conservation score ranged from 92,437 to 3,843,983 (Figure 4a), and the number of detected conserved sites ranged from 16,559 to 131,820 (Figure 4b and Supplemental Data S11). The LAST default parametrization led to detection of 98,193 conserved sites. The LAST strict parametrization led to detection of 113,253 conserved sites, corresponding to a 15.35% increase compared with the default (1.61% increase in CDS region, 75.75% increase in noncoding functional region). In line with this result, the parameter combination LAST relaxed elevated the number of detected conserved sites by 19.77% (−4.43% in CDS region, 114.31% increase in noncoding functional region) (Figure 4b, Supplemental Table S11, and Supplemental Data S11). We found a similar substantial increase in the detectable conserved sites in the BOP clade (Supplemental Figure S8 and Supplemental Data). This supports the conclusion that the alignment parametrization is applicable across a broad range of species. The mean Pearson's correlation (*r*) in conservation scores between the PACMAD and BOP clades in syntenic regions was moderate (*r* = 0.25) with limited variability between alignment parameter combinations (Supplemental Figure S9). Consistent with the comparison of alignment rates between Cactus and the *msa_pipeline* parametrized with LAST relaxed, both methods performed similarly well at facilitating detection of conservation (Figure 4). Taken together, these results suggest that *msa_pipeline* is a flexible interspecies alignment solution producing similar alignment rates to the state‐of‐the‐art Cactus aligner. By parametrizing the LAST aligner to be more strict or relaxed, users can trade off the amount of non‐CDS aligned with alignment precision. In particular, the HOXD70 substitution matrix combined with a relatively low gap‐open penalty (LAST relaxed) is preferable to the default LAST substitution matrix and gap‐open penalty for detection of plant conserved noncoding elements.

## 4 CONCLUSIONS

The *msa_pipeline* leverages existing tools to provide a practical solution for rapid multiple alignment of genomes with minimal user effort. For divergent plant genomes, different repeat masking approaches had limited effect on the alignment rate, but reduction of gap‐related alignment penalties boosted alignment rates of noncoding functional elements. We anticipate that the accelerating pace of genome sequencing and assembly will generate rich resources for genome‐scale multiple alignments that drive biological discovery in plants.

## AUTHOR CONTRIBUTIONS

Yaoyao Wu: Conceptualization; Formal analysis; Visualization; Writing‐original draft; Writing‐review & editing. Lynn Johnson: Formal analysis; Methodology; Software; Writing‐review & editing. Baoxing Song: Methodology; Writing‐review & editing. Cinta Romay: Funding acquisition; Project administration; Supervision; Writing‐review & editing. Michelle Stitzer: Methodology; Writing‐review & editing. Adam Siepel: Conceptualization; Funding acquisition; Supervision; Writing‐review & editing. Edward Buckler: Conceptualization; Funding acquisition; Supervision; Writing‐review & editing. Armin Scheben: Conceptualization; Formal analysis; Methodology; Software; Writing‐original draft; Writing‐review & editing.

## CONFLICT OF INTEREST

The authors declare no conflict of interest.

## Supporting information

Supplemental Figure S1. Impact of repeat masking methods on alignment of functional genomic regions in the BOP grass clade.
Supplemental Figure S2. The mini‐genome construction pipeline.
Supplemental Figure S3. Comparison of genome size of the mini‐genome sequences syntenic to the 100 gene region on sorghum chromosome 1 (Supplemental Data S1).
Supplemental Figure S4. Comparison of the query‐to‐reference pairwise alignment rate for the whole genome and the sorghum chromosome 3 mini‐genome in 30 grass species.
Supplemental Figure S5. Multiple alignment performance of 750 LAST parameter combinations for regions syntenic to the Oryza sativa mini‐genome sequence (Supplemental Data S2) in the BOP grass clade.
Supplemental Figure S6. Multiple alignment performance of 750 LAST parameter combinations for regions syntenic to the sorghum chromosome 1 mini‐genome (Supplemental Data S1) with 750 LAST parameter combinations in the PACMAD grass clade.
Supplemental Figure S7. Whole genome pairwise alignment metrics of the 13 PACMAD species aligned to the maize reference using three parametrizations of LAST.
Supplemental Figure S8. Conservation scoring based on multiple alignments generated with 750 different LAST parameter combinations for BOP clade.
Supplemental Figure S9. Correlation of GERP conservation scores between PACMAD and BOP clades for 750 LAST parameter combinations in different genomic regions.
Supplemental Table S1. Computational resources used by *msa_pipeline* for multiple alignment using different species sets and masking approaches.
Supplemental Table S2. Comparison of three masking methods (KMER, RED, and RepeatMasker) in *Zea mays* functional regions.
Supplemental Table S3. Comparison of three masking methods (KMER, RED, and RepeatMasker) in *Oryza sativa* functional regions.
Supplemental Table S4. Statistical comparison of F_1_ alignment score distribution for 13 PACMAD grass genomes masked using different methods (Supplemental Data S10).
Supplemental Table S5. Memory usage and runtime for *msa_pipeline* using RepeatMasker (RM) and RED with soft‐masking (sm) and hard‐masking (rm).
Supplemental Table S6. LAST alignment parameter ranges used for exploration of the parameter space. Step size of parameter value ranges shown in parentheses.
Supplemental Table S7. Alignment metrics in different genomic regions of mini‐genome 1 (100‐gene region on sorghum chr 3) based on 750 tested LAST parameter combinations.
Supplemental Table S8. Parameter settings of the LAST aligner at a default setting and at the relaxed and strict settings.
Supplemental Table S9. Alignment metrics comparison for the Cactus aligner and three parameter combinations for the LAST aligner in the PACMAD clade.
Supplemental Table S10. Comparison of pairwise plant genome alignments for the Cactus aligner and three parameter combinations for the LAST aligner.
Supplemental Table S11. Conserved sites comparison in the PACMAD clade for the Cactus aligner and three parameter combinations for the LAST aligner.

## Data Availability

Data used in this study can be found in the Supplemental Material for this publication (https://doi.org/10.6084/m9.figshare.17283806.v2). The *msa_pipeline* code is available at https://bitbucket.org/bucklerlab/msa_pipeline/.
