# Supplementary material for: A multiple alignment workflow shows the effect of repeat masking and parameter tuning on alignment in plants
Source: Plant Genome. 2022 Apr 13;15(2):e20204. doi: 10.1002/tpg2.20204 (PMC12807243; doi:10.1002/tpg2.20204)
Supplement: Supplementary file 1 [file TPG2-15-e20204-s001.pdf]

Supplemental Materials: A multiple alignment workflow shows the effect of repeat masking and parameter tuning on alignment in plants

Yaoyao Wu[1,2+*], Lynn Johnson[1+], Baoxing Song[1], Cinta Romay[1], Michelle Stitzer[1,5], Adam Siepel[4], Edward Buckler[1,3,5], Armin Scheben[4*]

[1]Institute for Genomic Diversity, Cornell University, Ithaca, NY 14853, USA
[2]Agricultural Genomics Institute at Shenzhen, Chinese Academy of Agricultural Sciences, Shenzhen, China
[3] Agricultural Research Service, US Department of Agriculture, Ithaca, NY 14853, USA
[4] Simons Center for Quantitative Biology, Cold Spring Harbor Laboratory, Cold Spring Harbor, NY 11724, USA
[5] Department of Molecular Biology and Genetics, Cornell University, Ithaca, NY 14853. USA

+These authors contributed equally

Supplemental results

Impact of repeat masking on alignment

Compared to KMER, RepeatMasker masked an additional 22.76% (whole genome), 28.89% (CDS), 30.28% (open chromatin) and 38.98% (non-coding functional) in maize (Figure 2A). And when compared to RED, RepeatMasker masked 10.25%, 20.08%, 29.28% and 24.97% more in the whole genome, CDS region, open-chromatin and non-coding functional region (Figure 2A). RepeatMasker thus had the highest masking rate but for many species, it also masked the highest proportion of functional elements occurring in coding and open chromatin sequence, and non-coding functional region (Figure 2A). KMER displayed the most favorable trade-off between the masking rate and the rate of masked coding and open chromatin sequence. This difference between RepeatMasker and KMER was not specific to maize, it was noticeable in most grass genomes (Figure 2B and Supplemental Figure S1). However, KMER performance declined in cases where the genome was poorly assembled, as is the case for *Dichanthelium oligosanthes* and *Eragrostis tef*.

To further investigate the impact of masking on pairwise alignment, we analyzed the alignment recall, precision and F_1 score for CDS, open chromatin regions, non-coding functional regions and all functional regions (Figure 2C). Using pairwise alignments of 32 grass species, the average F_1 scores were 0.40 (KMER), 0.35 (RED), 0.29 (RepeatMasker), and 0.32 (unmasked). This showed that KMER produces the highest F_1 score and that hard-masked alignment performs similarly to soft-masked alignment (Figure 2, Supplemental Figure S1) but with a significant speed-up (Supplemental Table S1). For the multiple alignment of all species in the PACMAD clade, the F_1 is 0.4309 (KMER), 0.3919 (RED), 0.3203 (RepeatMasker), and 0.3542 (unmasked) for functional

regions. Although alignments with KMER masking received the highest F_1 score in both the PACMAD and BOP clade (Figure S1D), the repeat masking approach only had a substantial impact on alignment rates when using hard-masking. These results suggest that the *msa_pipeline* is not sensitive to the repeat-masking approach for soft masked genomes.

Supplemental figures and tables

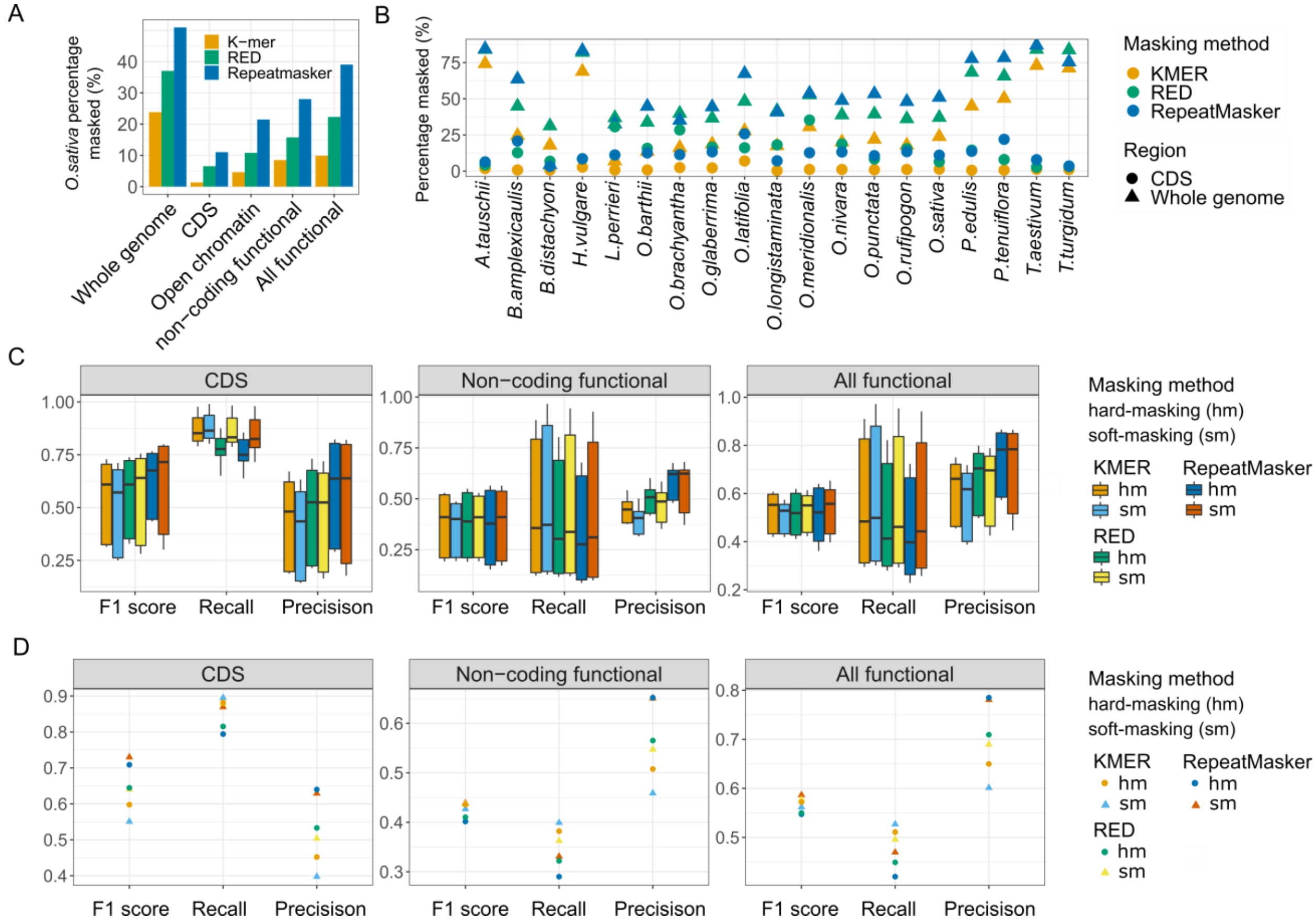

Supplemental Figure S1. Impact of repeat masking methods on alignment of functional genomic regions in the BOP grass clade. (A) The masking rate for the whole genome, CDS, open chromatin, non-coding functional and all functional regions using three different masking methods in rice. (B) The masking rate for the whole genome (triangle) and for CDS regions (circle) in 19 species of the BOP clade. (C) Boxplots of pairwise alignment performance of each species in the BOP clade against the rice genome using different genome masking methods. Performance is measured by precision, recall and F_1 score (harmonic mean of precision and recall). (D) Multiple alignment performance in the BOP clade using different genome masking methods.

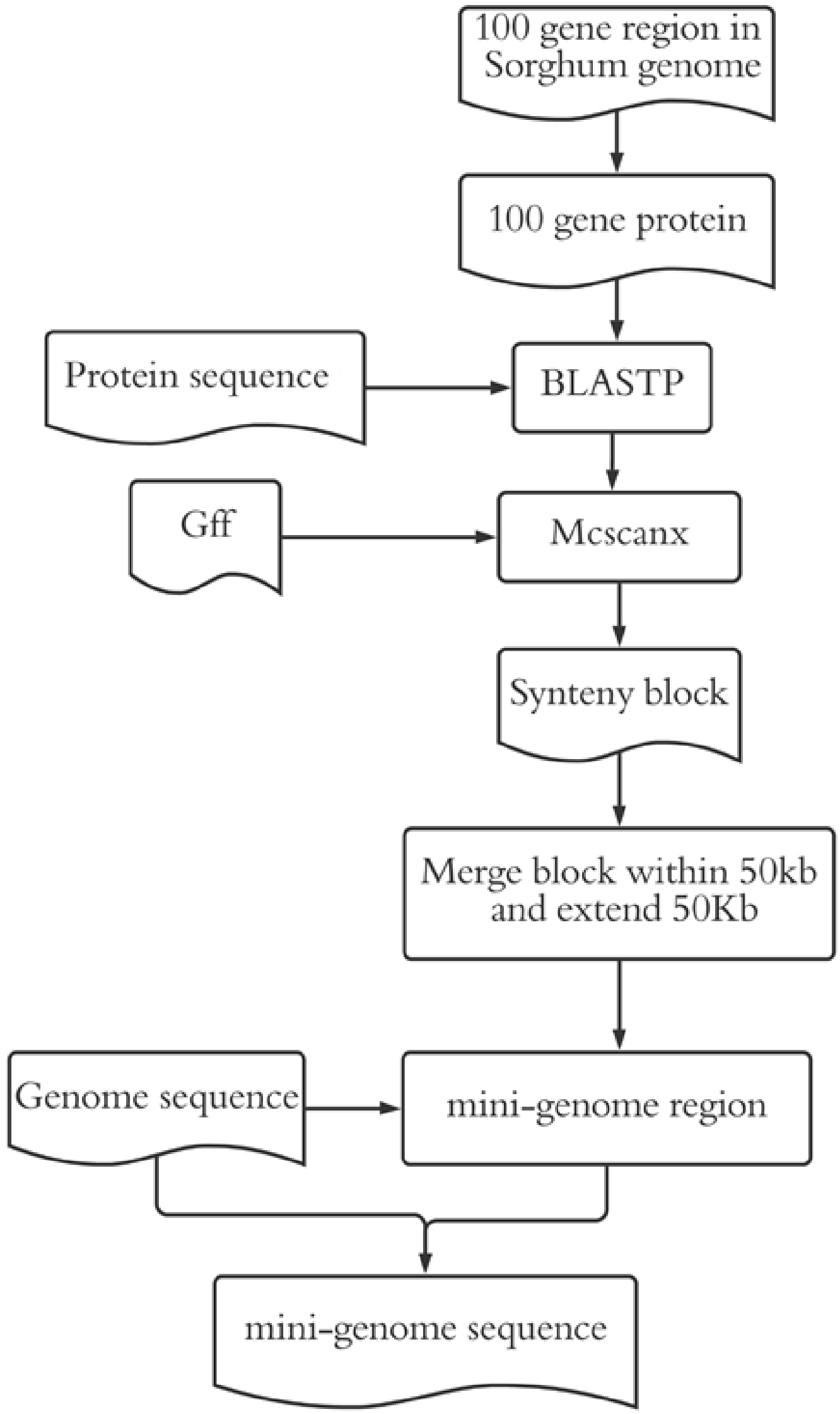

Supplemental Figure S2. The mini-genome construction pipeline. Starting with a genomic region covering 100 genes in *Sorghum bicolor*, syntenic blocks in 31 other grass species were identified using MCScanX and then extracted to generate a syntenic sequence, here referred to as a mini-genome, for each species.

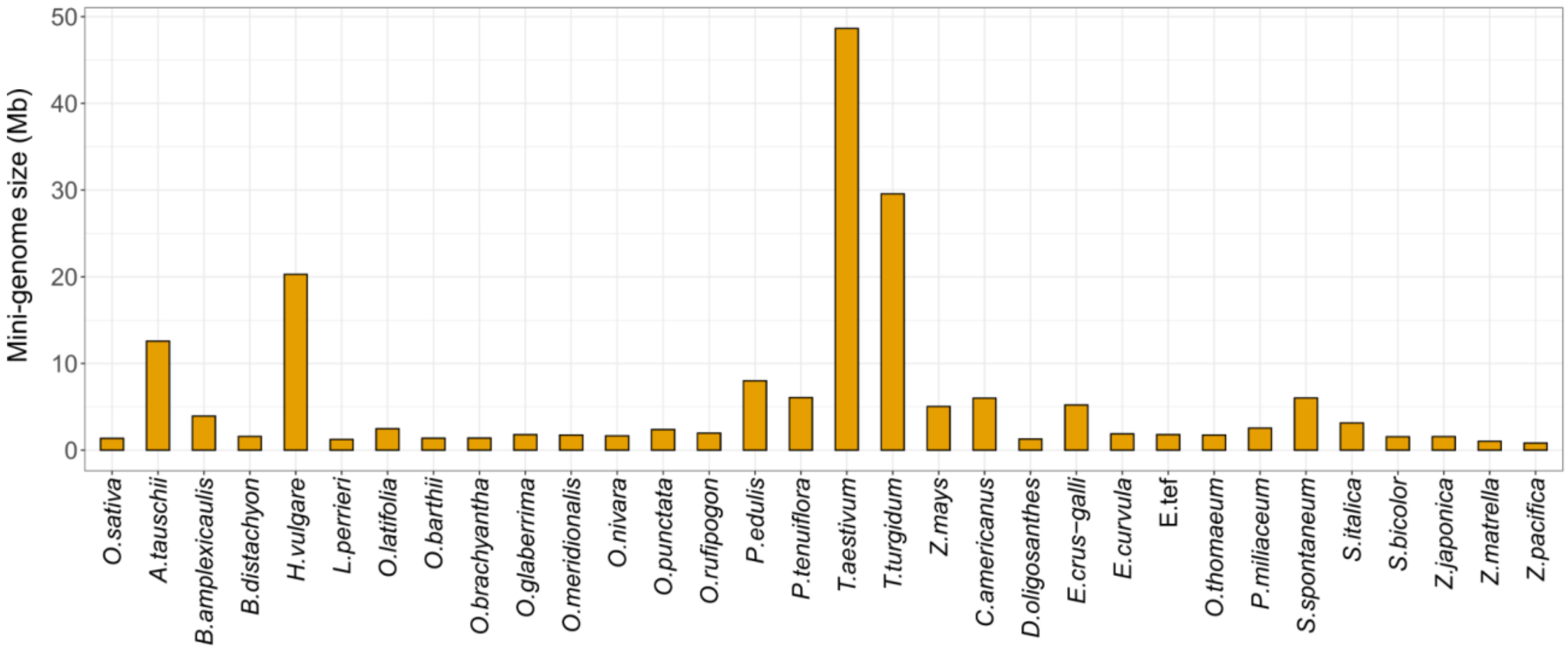

Supplemental Figure S3. Comparison of genome size of the mini-genome sequences syntenic to the 100 gene region on sorghum chromosome 1 (Supplemental Data S1).

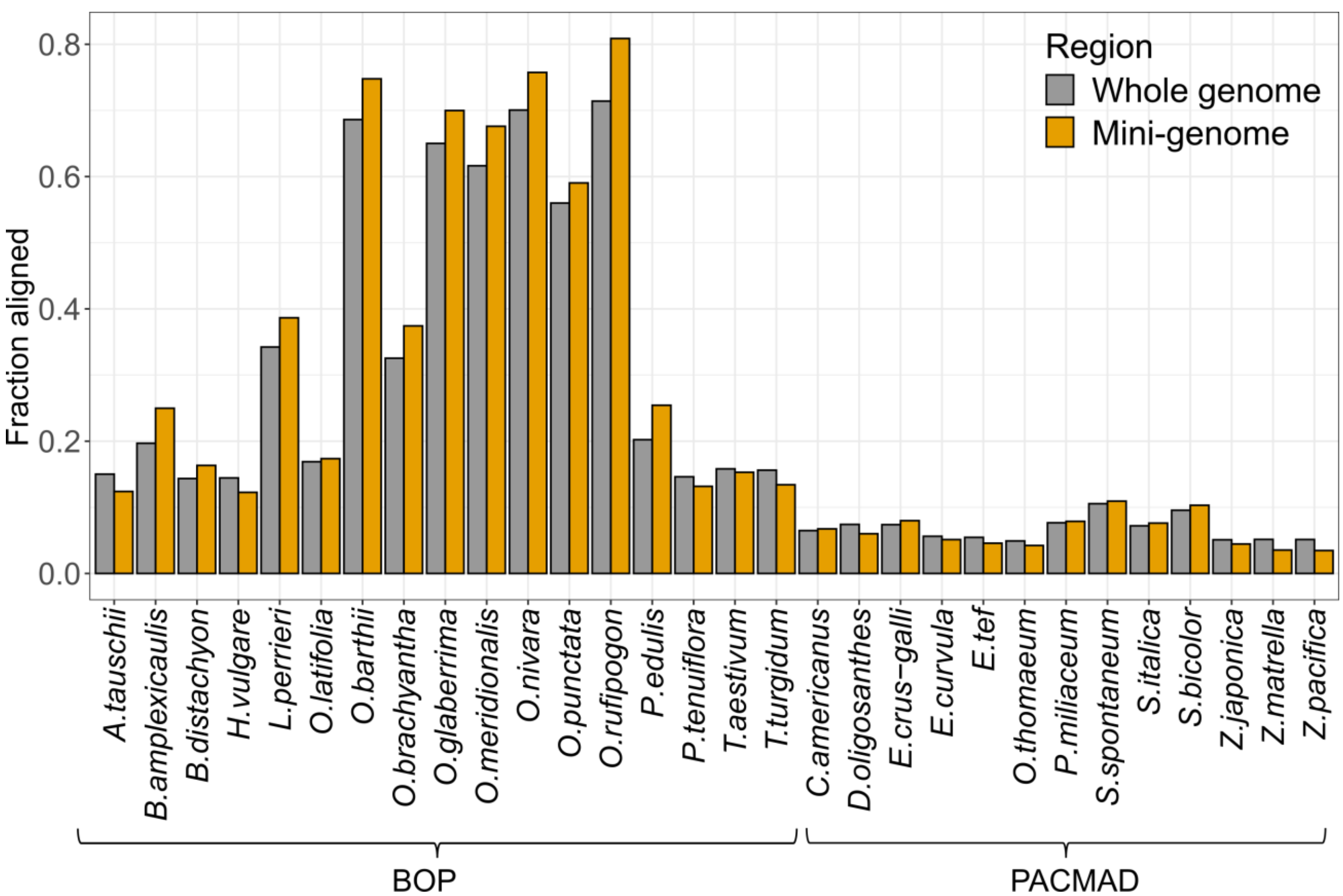

Supplemental Figure S4. Comparison of the query-to-reference pairwise alignment rate for the whole genome and the sorghum chromosome 3 mini-genome in 30 grass species. The grey bar is the alignment rate of each species aligned to reference species (*Zea mays* for PACMAD grass clade, *Oryza sativa* for BOP grass clade). The yellow bar is the alignment rate of each mini-

genome aligned to the reference mini-genome. Alignment rates are consistent between the mini-genome and whole genome, suggesting that the mini-genome is representative of the wider genome.

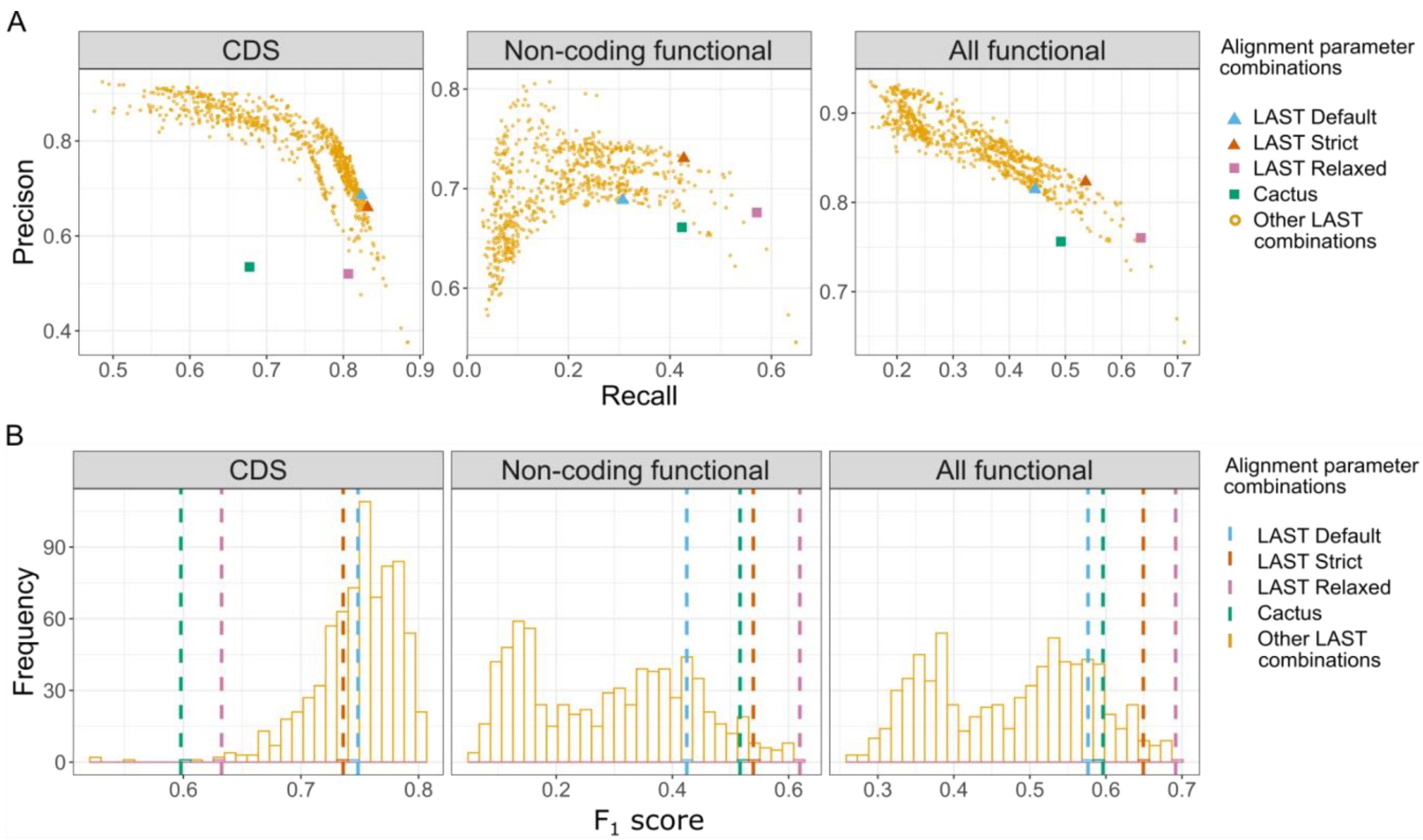

Supplemental Figure S5. Multiple alignment performance of 750 LAST parameter combinations for regions syntenic to the *Oryza sativa* mini-genome sequence (Supplemental Data S2) in the BOP grass clade. Tested parameter combinations are compared to the alignment performance of default LAST parameters and the Cactus aligner based on (A) recall and precision as well as the (B) F_1 score (harmonic mean of precision and recall).

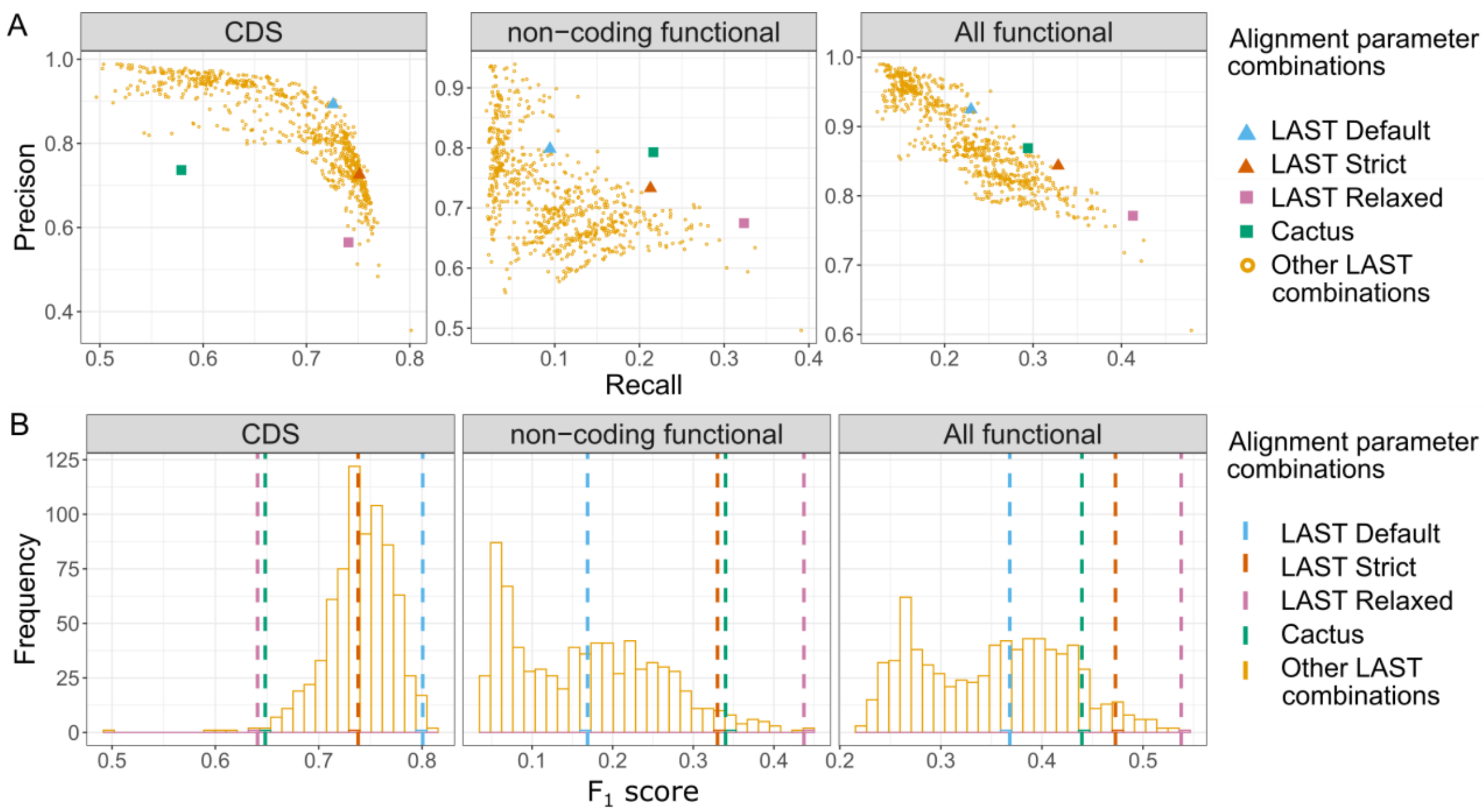

Supplemental Figure S6. Multiple alignment performance of 750 LAST parameter combinations for regions syntenic to the sorghum chromosome 1 mini-genome (Supplemental Data S1) with 750 LAST parameter combinations in the PACMAD grass clade. Tested parameter combinations are compared to the alignment performance of default LAST parameters and the Cactus aligner based on (A) recall and precision as well as the (B) F_1 score (harmonic mean of precision and recall).

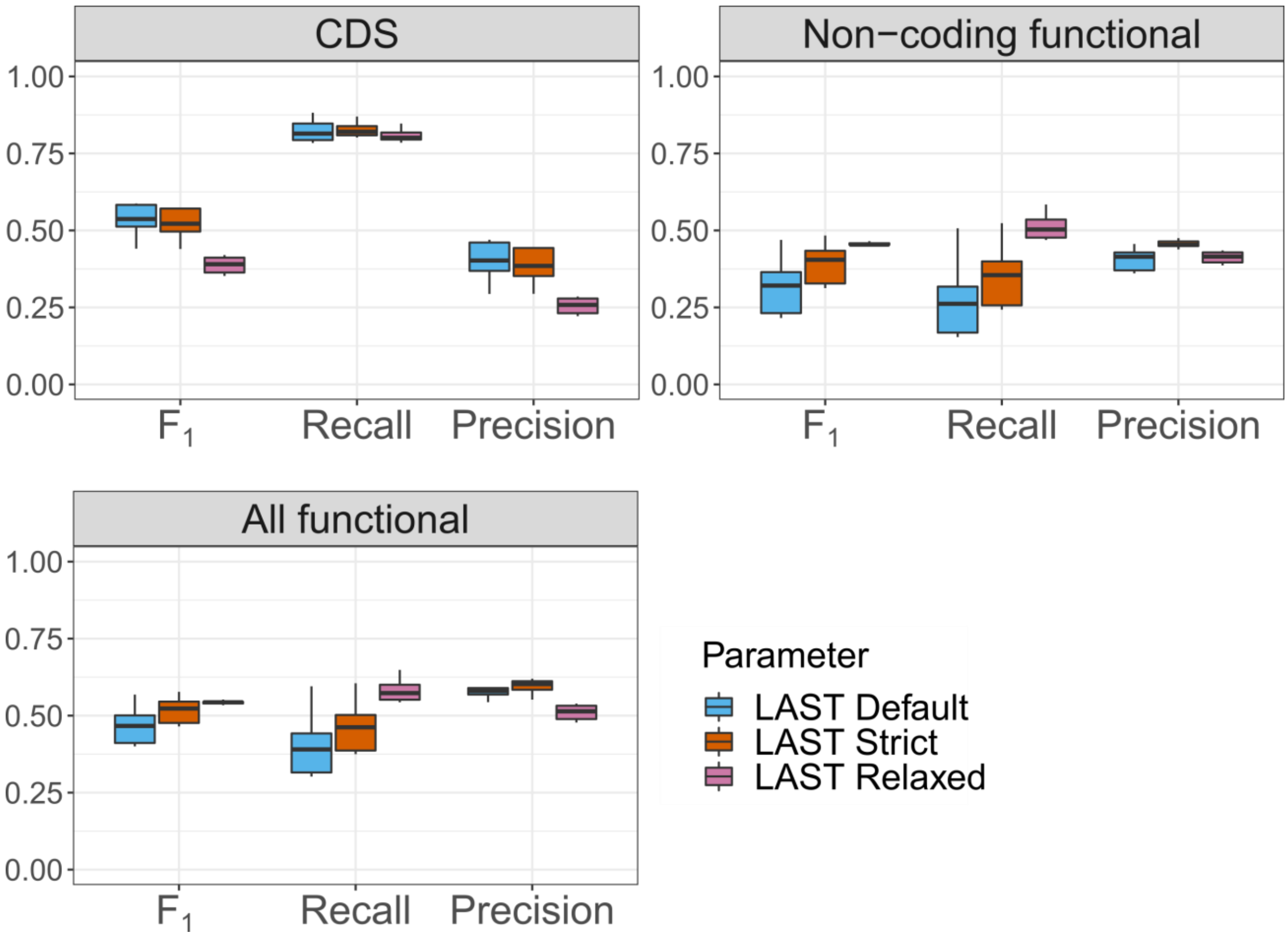

Supplemental Figure S7. Whole genome pairwise alignment metrics of the 13 PACMAD species aligned to the maize reference using three parametrizations of LAST. Underlying data is available in the Supplemental Data.

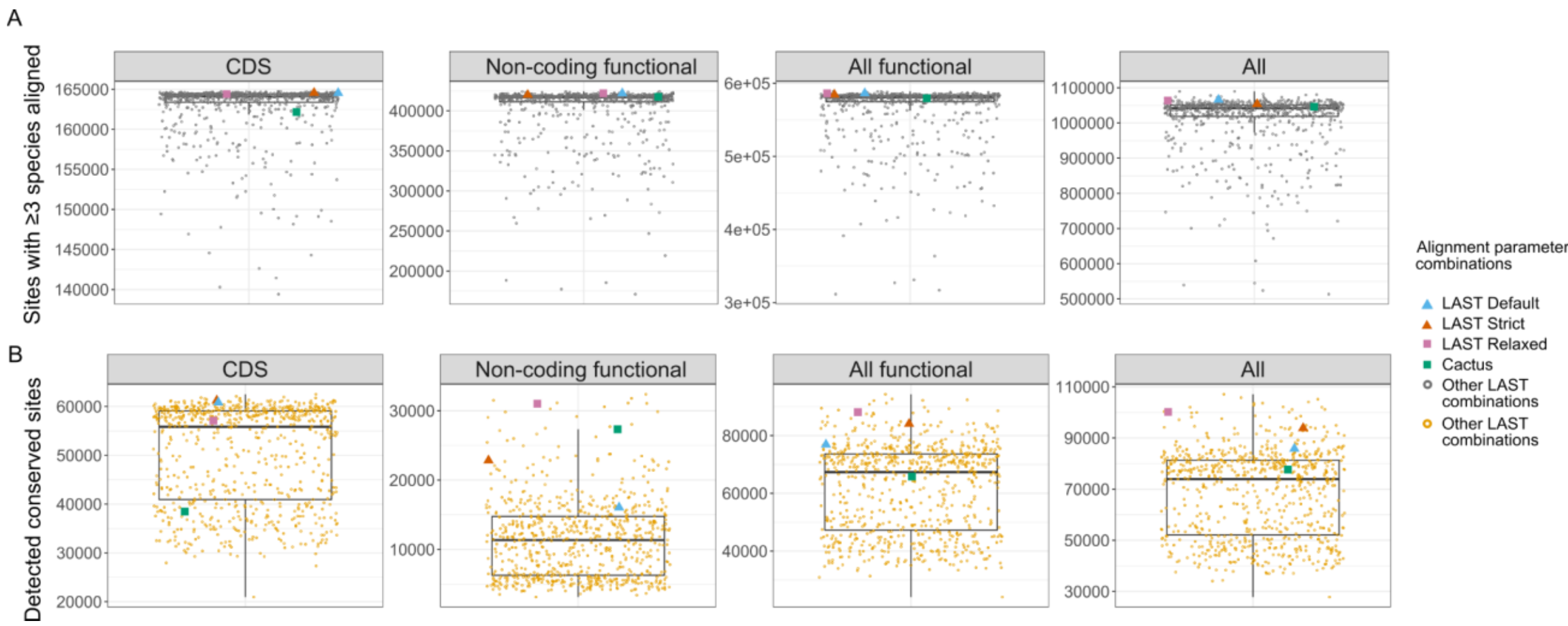

Supplemental Figure S8. Conservation scoring based on multiple alignments generated with 750 different LAST parameter combinations for BOP clade.

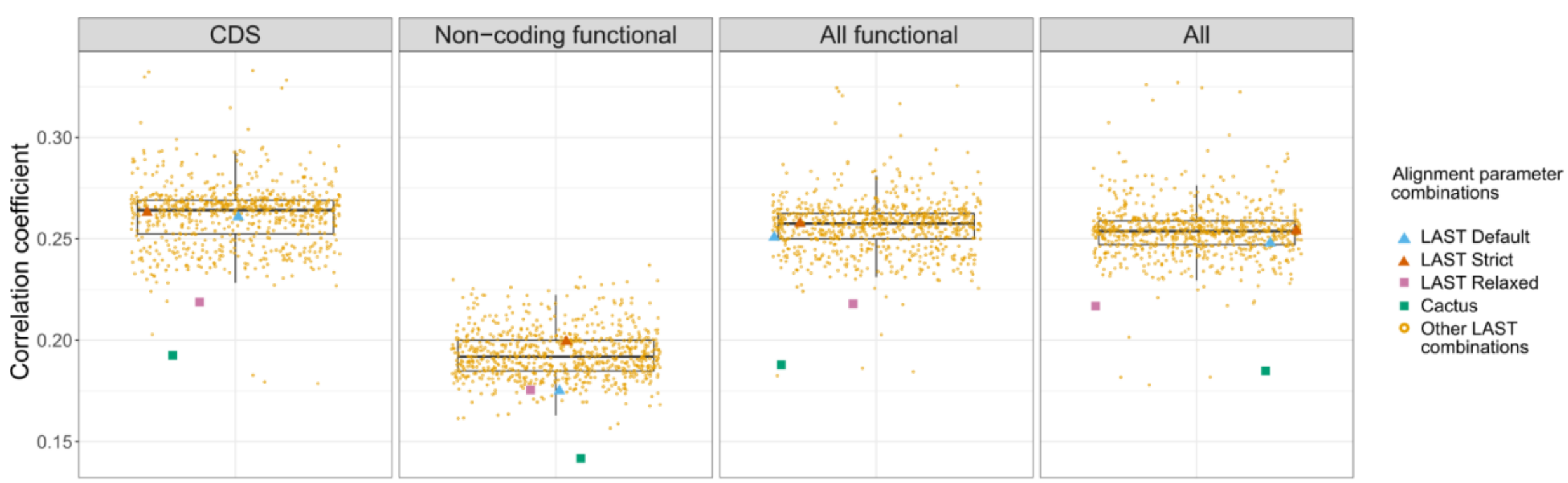

Supplemental Figure S9. Correlation of GERP conservation scores between PACMAD and BOP clades for 750 LAST parameter combinations in different genomic regions.

Supplemental Table S1. Computational resources used by *msa_pipeline* for multiple alignment using different species sets and masking approaches. A comparison estimated resource requirements for the Cactus multiple aligner highlights the relative speed and low memory use of the *msa_pipeline*.

Clade	# Species	Masking	Runtime with 12 threads (h)	Max memory (Gb)
PACMAD	14	soft	31.8	15.8
PACMAD	14	hard	27.8	16.8
BOP	19	soft	51.0	22.1
BOP	19	hard	45.4	22.7

Supplemental Table S2. Comparison of three masking methods (KMER, RED, and RepeatMasker) in *Zea mays* functional regions.

Region	Genome size (bp)	Masked (bp)			Masked percentage		
		KMER	*RED*	*RepeatMasker*	*KMER*	*RED*	*Repeat Masker*
Whole genome	2,135,083,061	1,420,031,158	1,687,073,931	1,895,632,680	66.51%	79.02%	88.78%
CDS	48,439,693	1,970,871	6,236,626	15,964,902	4.07%	12.88%	32.96%
Open chromatin	9,359,052	77,819	171,552	2,911,939	0.83%	1.83%	31.11%
Non-coding functional	157,282,892	23,623,428	45,664,878	84,899,017	15.02%	29.03%	53.98%
All functional	205,722,585	25,594,299	51,901,504	100,863,919	12.44%	25.23%	49.03%

Supplemental Table S3. Comparison of three masking methods (KMER, RED, and RepeatMasker) in *Oryza sativa* functional regions.

Region	Genome size (bp)	Masked (bp)			Masked percentage		
		KMER	*RED*	*RepeatMasker*	*KMER*	*RED*	*Repeat Masker*
Whole genome	375,049,285	89,420,319	138,926,384	190,998,285	23.84%	37.04%	50.93%
CDS	34,424,450	469,841	2,249,860	3,798,942	1.36%	6.54%	11.04%
Open chromatin	7,787,654	359,464	842,284	1,672,469	4.62%	10.82%	21.48%
Non-coding functional	99,293,331	8,447,968	15,664,311	27,808,668	8.51%	15.78%	28.01%
All functional	133,717,781	8,917,809	17,914,171	31,607,610	6.67%	13.40%	23.64%

Supplemental Table S4. Statistical comparison of F_1 alignment score distribution for 13 PACMAD grass genomes masked using different methods (Supplemental Data S10). Hard-masking (hard) and soft-masking (soft) may impact alignment and were also compared.

**Method 1**	**Method 2**	**Student's t-test p-value**	**One-sided test**
KMER-hard	RED-hard	0.07215	greater
KMER-hard	RM-hard	0.0001105	greater
RED-hard	RM-hard	0.007751	greater
KMER-hard	KMER-soft	0.7471	less
RED-hard	RED-soft	0.2351	less
RM-hard	RM-soft	0.1772	less

Supplemental Table S5. Memory usage and runtime for *msa_pipeline* using RepeatMasker (RM) and RED with soft-masking (sm) and hard-masking (rm).

Clade	Number of species	Masking	Max memory (Gb)	CPU hours	CPU hours longest species	CPU hours ROAST	Runtime (h) with 12 CPU
PACMAD	14	RMsm	10.1	13.7	2.6	4.6	7.2
PACMAD	14	RMrm	10.4	13.1	2.6	4.0	6.7
PACMAD	14	REDsm	12.1	35.6	3.9	17.8	21.8
PACMAD	14	REDrm	14.8	30.1	3.4	13.6	17.0
BOP	19	RMsm	16.5	25.4	2.7	14.4	17.2
BOP	19	RMrm	16.5	21.7	2.6	10.8	13.4
BOP	19	REDrm	22.0	52.8	6.0	26.7	32.7
BOP	19	REDsm	19.5	61.6	6.0	35.2	41.2

Supplemental Table S6. LAST alignment parameter ranges used for exploration of the parameter space. Step size of parameter value ranges shown in parentheses. See Supplemental Data for RETRO SIMPLE and RETRO custom matrices.

	**Default parameters for two reference penalty matrices**		**Parameter ranges for five penalty matrices**				
LAST Parameter	*HOXD70*	*1:1:1:7:1*	*1:1:1:7:1*	*2:1:2:16:1*	*RETRO SIMPLE*	*RETRO*	*HOXD70*
-R	10	10	10,11	10,11	10,11	10,11	10,11
-c	-c	-	-c	-c	-c	-c	-c
-u	MAM8	YASS	YASS,MAM8	YASS,MAM8	YASS,MAM8	YASS,MAM8	YASS,MAM8
-a	400	7	3-16 (1)	6-32 (2)	3-16 (1)	300-1600 (100)	300-1600 (100)
-b	30	1	1-3 (1)	1-3 (1)	1-3 (1)	20-300 (10)	20-300 (10)
-e	4000	32	30-60 (10)	60-120 (20)	30-60 (10)	3000-5000 (1000)	3000-5000 (1000)
-m	100	10	50-150 (50)	50-150 (50)	50-150 (50)	50-150 (50)	50-150 (50)
-y	-	9	9-50 (5)	15-60 (5)	9-50 (5)	1000-2000 (200)	1000-2000 (200)
-x	-	25	20-100 (10)	30-150 (10)	20-100 (10)	1500-2500 (250)	1500-2500 (250)
-d	-	27	e*0.7	e*0.7	e*0.7	e*0.7	e*0.7
-p	HOXD70	-	-	custom matrix	custom matrix	custom matrix	HOXD70
-u	2	2	1-3 (1)	1-3 (1)	1-3 (1)	1-3 (1)	1-3 (1)

Supplemental Table S7. Alignment metrics in different genomic regions of mini-genome 1 (100-gene region on sorghum chr 3) based on 750 tested LAST parameter combinations.

	**Recall**			**Precision**			F_1 **score**		
Region	*min*	*max*	*mean*	*min*	*max*	*mean*	*min*	*max*	*mean*
Coding	0.4870	0.7824	0.6800	0.4730	0.9870	0.8949	0.5896	0.8234	0.7673
Coding + non-coding functional	0.1143	0.4737	0.2264	0.7211	0.9887	0.9339	0.2049	0.5778	0.3578
Non-coding	0.0152	0.3931	0.1057	0.6280	0.9272	0.8221	0.0298	0.4907	0.1782

Supplemental Table S8. Parameter settings of the LAST aligner at a default setting and at the relaxed and strict settings. The relaxed and strict settings were selected for divergent interspecies genome alignment from 750 parameter combinations tested in this study.

**Parameter**	**Parameter meaning**	**Default**	**Relaxed**	**Strict**
-R	repeat-marking options (10: do nothing, 11: carry out additional simple repeat masking with tantan)	10	11	10
-c	Exclude masked sequence from initial matches	no	yes	yes
-u	seeding scheme, sets matrix and sets penalty scores for lastal	YASS	YASS	YASS
-a	Gap/Insertion existence cost	7	300	700
-b	Gap extension cost	1	20	20
-e	Minimum alignment score	32	5000	5000
-m	Initial match number	10	150	50
-y	Maximum score drop for gapless alignments	9	1800	1400
-x	This option makes lastal extend gapped alignments twice. First, it extends gapped alignments with a maximum score drop of x	25	1750	1750
-d	Minimum score for gapless alignments. Can be set to equal -e value	27	3500	3500
-p	Mismatch matrix for alignment extension	equal mismatch weight matrix	HOXD70	HOXD70
-u	Specify treatment of lowercase letters when extending alignments (1:Mask them for gapless but not gapped extensions; 2: Mask them for gapless but not gapped extensions, and then discard alignments that lack any segment with score ≥ e when lowercase is masked)	2	1	1

Supplemental Table S9. Alignment metrics comparison for the Cactus aligner and three parameter combinations for the LAST aligner in the PACMAD clade. Alignments were conducted on mini-genome 1 (100-gene region on sorghum chr 3).

	**All functional region**			**Coding region**			**Non-coding functional region**		
**Alignment**	*F_1 score*	*recall*	*precision*	*F_1 score*	*recall*	*precision*	*F_1 score*	*recall*	*precision*
Cactus	0.5472	0.4040	0.8478	0.7243	0.7637	0.6888	0.4404	0.3083	0.7706
LAST default	0.4309	0.2823	0.9095	0.7952	0.7483	0.8485	0.2652	0.1583	0.8166
LAST strict	0.5037	0.3483	0.9098	0.7825	0.7484	0.8199	0.3762	0.2418	0.8469
LAST relaxed	0.5685	0.4270	0.8500	0.7013	0.7337	0.6717	0.4795	0.3454	0.7836

Supplemental Table S10. Comparison of pairwise plant genome alignments for the Cactus aligner and three parameter combinations for the LAST aligner. The tomato reference genome (GenBank: GCA_000188115.3) and the potato genome (Genbank: GCA_000226075.1) were downloaded from GenBank. Approximate (Appr.) measures of recall and precision were calculated as described in the Methods.

**Query (Q) and reference (R) species**		**All functional regions**			**Coding regions**			**Non-coding functional regions**		
	**Alignment**	**F1 score**	**Appr. recall**	**Appr. precision**	**F1 score**	**Appr. recall**	**Appr. precision**	**F1 score**	**Appr. recall**	**Appr. precision**
R: Maize Q: Sorghum	Cactus	0.4428	0.32	0.72	0.51	0.521	0.493	0.363	0.26	0.61
	LAST default	0.5663	0.564	0.57	0.47	0.872	0.324	0.4627	0.47	0.456
	LAST strict	0.5746	0.568	0.58	0.48	0.866	0.333	0.4735	0.48	0.4708
	LAST relaxed	0.5592	0.632	0.5	0.38	0.856	0.243	0.472	0.56	0.4066
R: Sorghum Q: Maize	Cactus	0.5862	0.466	0.79	0.62	0.669	0.58	0.5074	0.4	0.705
	LAST default	0.6022	0.553	0.66	0.5	0.456	0.545	0.5669	0.84	0.4285
	LAST strict	0.612	0.556	0.68	0.58	0.837	0.449	0.5081	0.46	0.5673
	LAST relaxed	0.6185	0.65	0.59	0.47	0.851	0.325	0.5313	0.58	0.4894
R: Tomato Q: Potato	Cactus	0.4286	0.84	0.29	0.58	0.669	0.518	0.6476	0.71	0.5967
	LAST default	0.4815	0.84	0.34	0.21	0.961	0.118	0.4074	0.8	0.2728
	LAST strict	0.4878	0.832	0.35	0.22	0.956	0.122	0.4134	0.8	0.2793
	LAST relaxed	0.4917	0.815	0.35	0.22	0.929	0.125	0.4188	0.78	0.2861

Supplemental Table S11. Conserved sites comparison in the PACMAD clade for the Cactus aligner and three parameter combinations for the LAST aligner.

**Alignment**	**Conserved sites**	**Conserved coding sites**	**Conserved non-coding functional sites**
Cactus	124,199	68,752	43,895
LAST default	98,193	76,541	17,544
LAST strict	113,253	77,771	30,834
LAST relaxed	117,602	73,155	37,598
